# Supplementary material for: Muscular Fatigue and Quadriceps-to-Hamstring Ratio in Alpine Skiing in Women over 40 Years
Source: Int J Environ Res Public Health. 2023 Apr 12;20(8):5486. doi: 10.3390/ijerph20085486 (PMC10138889; doi:10.3390/ijerph20085486)
Supplement: Supplementary file 1 [file ijerph-20-05486-s001.zip › ijerph-2279652-supplementary.pdf]

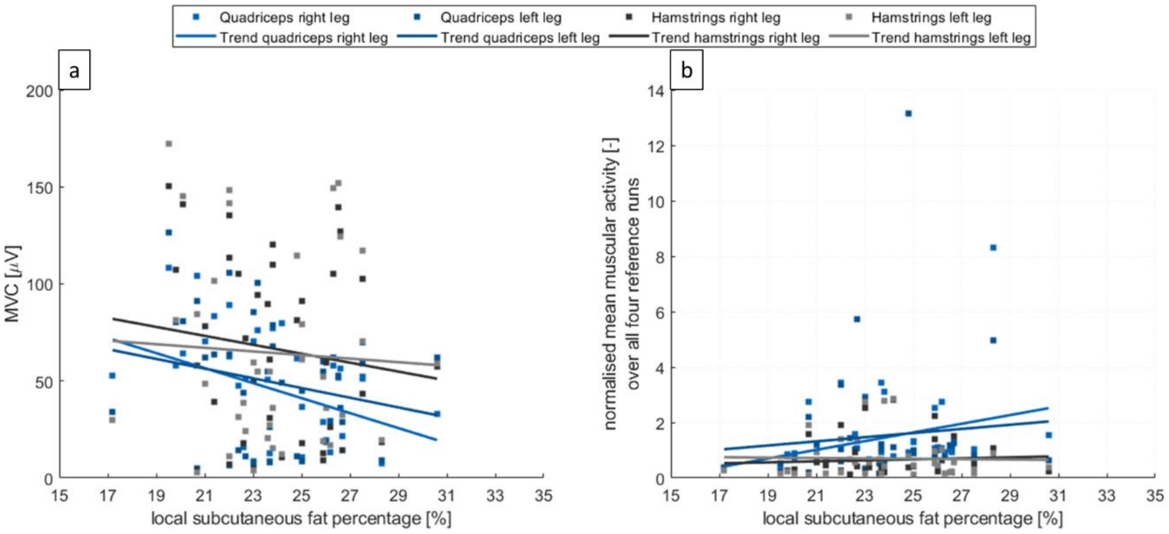

Figure S1. (**a**) The MVC values of the quadriceps and hamstring groups of all participants and the trendline with respect to the participants' local subcutaneous fat percentage. (**b**) The normalised mean muscular activity over all reference runs of the day for the four muscle groups and all participants with respect to the participants' local subcutaneous fat percentage.

Table S1. Descriptive statistics of muscular activity (normalised with the MVC) for the four muscle groups during the four different skiing tasks in the four reference runs; values state the mean value ± standard deviation. Time of reference run 1: 10 am; time of reference run 2: 12 pm; time of reference run 3: 1 pm time of reference run 4: 3 pm.

	Quadriceps		Hamstrings	
	right	left	right	left
Plow reference run 1	1.20 ± 1.14	1.14 ± 1.27	0.37 ± 0.42	0.43 ± 0.56
Plow reference run 2	1.34 ± 1.76	1.21 ± 1.39	0.38 ± 0.50	0,42 ± 0.52
Plow reference run 3	0.97 ± 1.06	1.04 ± 1.91	0.30 ± 0.30	0.33 ± 0.39
Plow reference run 4	1.22 ± 1.81	1.35 ± 2.77	0.39 ± 0.47	0.42 ± 0.44
Plow total	1.18 ± 1.37	1.18 ± 1.66	0.36 ± 0.40	0.40 ± 0.46
Skating step reference run 1	1.66 ± 1.28	1.58 ± 1.99	0.80 ± 0.87	0.73 ± 0.76
Skating step reference run 2	1.66 ± 1.60	1.51 ± 1.78	0.83 ± 0.95	0.81 ± 0.84
Skating step reference run 3	1.38 ± 1.26	1.73 ± 3.67	0.57 ± 0.53	0.74 ± 0.88
Skating step reference run 4	1.49 ± 2.04	1.85 ± 4.99	0.71 ± 0.87	0.73 ± 0.75
Skating step total	1.55 ± 1.44	1.67 ± 3.03	0.73 ± 0.75	0.75 ± 0.78
Small radius reference run 1	1.82 ± 1.64	1.78 ± 1.82	0.82 ± 0.96	0.87 ± 0.99
Small radius reference run 2	1.62 ± 1.85	1.56 ± 1.80	0,80 ± 0.95	0.88 ± 1.06
Small radius reference run 3	1.32 ± 1.23	1.59 ± 2.67	0.75 ± 1.14	0.84 ± 1.12
Small radius reference run 4	1.48 ± 1.70	1.80 ± 3.57	0.75 ± 0.90	0.85 ± 0.87
Small radius total	1.56 ± 1.54	1.68 ± 2.29	0.78 ± 0.91	0.86 ± 0.95
Medium radius reference run 1	1.80 ± 1.70	1.72 ± 1.67	0.79 ± 0.99	0.79 ± 1.02
Medium radius reference run 2	1.56 ± 1.66	1.50 ± 1.70	0.77 ± 1.04	0.88 ± 1.45
Medium radius reference run 3	1.27 ± 1.33	1.45 ± 2.58	0.67 ± 0.99	0.69 ± 0.96
Medium radius reference run 4	1.47 ± 1.97	1.71 ± 3.44	0.71 ± 0.88	0.81 ± 1.01
Medium radius total	1.52 ± 1.60	1.59 ± 2.21	0.73 ± 0.92	0.79 ± 1.08
Total reference run 1	1.62 ± 1.37	1.55 ± 1.59	0.70 ± 0.77	0.71 ± 0.77
Total reference run 2	1.55 ± 1.68	1.45 ± 1.61	0.70 ± 0.81	0.75 ± 0.85
Total reference run 3	1.23 ± 1.14	1.45 ± 2.68	0.57 ± 0.68	0.65 ± 0.75
Total reference run 4	1.42 ± 1.85	1.68 ± 3.67	0.64 ± 0.74	0.70 ± 0.68
Total	1.45 ± 1.46	1.53 ± 2.26	0.65 ± 0.71	0.70 ± 0.74

Table S2. Descriptive statistics of the quadriceps-to-hamstring-ratio for the left and right thigh during the four different skiing tasks in the four reference runs; values state the mean value ± standard deviation. Time of reference run 1: 10 am; time of reference run 2: 12 pm; time of reference run 3: 1 pm time of reference run 4: 3 pm.

	right	left
Plow reference run 1	6.30 ± 4.50	5.27 ± 3.67
Plow reference run 2	6.09 ± 4.62	5.82 ± 5.01
Plow reference run 3	5.59 ± 4.19	5.96 ± 8.16
Plow reference run 4	5.90 ± 4.61	5.36 ± 6.47
Plow total	5.97 ± 4.16	5.60 ± 5.47
Skating step reference run 1	4.46 ± 3.33	3.86 ± 2.71
Skating step reference run 2	4.20 ± 2.99	4.13 ± 3.89
Skating step reference run 3	3.99 ± 2.75	4.84 ± 6.20
Skating step reference run 4	4.42 ± 3.88	4.45 ± 7.04
Skating step total	4.27 ± 2.89	4.32 ± 4.67
Small radius reference run 1	4.00 ± 3.20	3.51 ± 2.86
Small radius reference run 2	3.78 ± 3.22	3.53 ± 3.33
Small radius reference run 3	3.76 ± 2.83	3.89 ± 4.47
Small radius reference run 4	3.87 ± 3.07	4.28 ± 7.44
Small radius total	3.85 ± 2.91	3.80 ± 4.29
Medium radius reference run 1	4.41 ± 3.54	4.18 ± 3.72
Medium radius reference run 2	4.06 ± 3.22	4.07 ± 4.75
Medium radius reference run 3	4.01 ± 3.00	4.63 ± 6.63
Medium radius reference run 4	4.02 ± 3.26	4.06 ± 5.38
Medium radius total	4.12 ± 3.11	4.24 ± 5.01
Total reference run 1	4.79 ± 3.45	4.20 ± 3.07
Total reference run 2	4.53 ± 3.33	4.39 ± 4.06
Total reference run 3	4.34 ± 3.02	4.83 ± 6.11
Total reference run 4	4.55 ± 3.53	4.54 ± 6.50
Total	4.55 ± 3.15	4.49 ± 4.76

Table S3. Descriptive statistics of the median frequency for the muscle groups of the left and right thigh during the four different skiing tasks in the four reference runs; values state the mean value ± standard deviation. Time of reference run 1: 10 am; time of reference run 2: 12 pm; time of reference run 3: 1 pm time of reference run 4: 3 pm.

	Quadriceps		Hamstrings	
	right	left	right	left
Plow reference run 1	77.22 ± 12.31	79.21 ± 9.46	80.93 ± 21.91	77.43 ± 13.97
Plow reference run 2	77.13 ± 12.67	75.59 ± 9.93	79.61 ± 21.22	78.08 ± 18.75
Plow reference run 3	74.88 ± 15.86	73.55 ± 16.89	70.90 ± 25.73	73.62 ± 29.20
Plow reference run 4	75.26 ± 14.05	74.85 ± 10.45	76.46 ± 20.26	76.99 ± 19.85
Plow total	76.12 ± 11.55	75.80 ± 9.26	76.97 ± 18.10	76.53 ± 17.04
Skating step reference run 1	73.00 ± 11.46	71.84 ± 11.82	67.68 ± 15.19	63.54 ± 11.59
Skating step reference run 2	71.14 ± 11.10	68.57 ± 12.45	67.53 ± 15.57	61.89 ± 13.23
Skating step reference run 3	65.69 ± 14.84	62.73 ± 14.75	59.40 ± 15.66	55.99 ± 14.71
Skating step reference run 4	67.01 ± 12.87	68.07 ± 13.20	64.92 ± 16.95	60.66 ± 15.70
Skating step total	69.21 ± 10.98	67.80 ± 11.43	64.88 ± 14.12	60.52 ± 12.13
Small radius reference run 1	70.72 ± 7.81	70.77 ± 7.00	68.55 ± 17.12	66.00 ± 13.22
Small radius reference run 2	67.86 ± 10.84	67.35 ± 10.75	66.07 ± 20.20	64.81 ± 17.31
Small radius reference run 3	64.03 ± 12.17	63.62 ± 12.53	58.26 ± 17.58	57.82 ± 16.51
Small radius reference run 4	64.03 ± 12.27	63.28 ± 11.75	61.84 ± 16.20	60.64 ± 18.00
Small radius total	66.66 ± 9.32	66.25 ± 9.52	63.68 ± 15.81	62.32 ± 14.68
Medium radius reference run 1	71.67 ± 7.73	71.08 ± 7.44	66.76 ± 17.23	67.00 ± 12.50
Medium radius reference run 2	67.85 ± 12.27	66.89 ± 10.92	66.32 ± 19.69	64.17 ± 15.65
Medium radius reference run 3	64.65 ± 12.67	63.85 ± 12.91	59.30 ± 16.17	59.28 ± 15.72
Medium radius reference run 4	64.58 ± 12.21	63.41 ± 11.51	63.43 ± 16.63	61.15 ± 17.30
Medium radius total	67.19 ± 9.92	66.31 ± 9.14	63.95 ± 15.28	62.90 ± 13.65
Total reference run 1	73.15 ± 8.26	73.23 ± 6.84	70.98 ± 14.04	68.49 ± 10.15
Total reference run 2	70.99 ± 10.11	69.60 ± 9.14	69.88 ± 16.25	67.24 ± 13.00
Total reference run 3	67.31 ± 11.48	65.94 ± 11.71	61.96 ± 15.82	61.68 ± 14.62
Total reference run 4	67.72 ± 10.95	67.40 ± 9.84	66.66 ± 14.33	64.86 ± 14.27
Total	69.79 ± 9.15	69.04 ± 8.18	67.37 ± 13.31	65.57 ± 11.53
